# Perfluoroalkyl substances in Baltic fish – the risk to consumers

**DOI:** 10.1007/s11356-023-26626-w

**Published:** 2023-04-03

**Authors:** Szczepan Mikolajczyk, Malgorzata Warenik-Bany, Marek Pajurek

**Affiliations:** Radiobiology Department, National Veterinary Research Institute, NRL for Halogenated POPs (PCDD/Fs, PCBs and PBDE) in Food and Feed, 57 Partyzantow Avenue, 24-100, Pulawy, Poland

**Keywords:** Fish, Baltic, PFASs, Dietary intake

## Abstract

Perfluoroalkyl substances (PFASs) were investigated in five Baltic fish species (sprat, herring, salmon, trout, and cod). Each species’ median lower bound (LB) concentration of ∑14 PFASs was as follows: in sprat it was 3.54 µg/kg wet weight (w.w.), in cod 2.15 µg/kg w.w., in salmon 2.10 µg/kg w.w., in trout 2.03 µg/kg w.w. and in herring 1.74 µg/kg w.w.. Regarding the species’ median LB of ∑4 PFASs (perfluorooctane sulfonic acid (PFOS), perfluorooctanoic acid (PFOA), perfluorononanoic acid (PFNA), and perfluorohexane sulfonic acid (PFHxS)), sprat was the most contaminated with 2.90 µg/kg w.w. and herring was the least with 1.17 µg/kg w.w.. Among all PFASs, PFOS was found in the highest concentrations (range 0.04–9.16 µg/kg w.w.) and the percentage share in the total concentration of ∑14 PFASs was between 56 and 73%. The average proportion of linear PFOS (L-PFOS) in the total PFOS (branched and linear) was the highest in salmon at 89% and trout at 87%, and in the other three species it ranged from 75 to 80%. Different consumption scenarios were assumed and the intake of PFASs was calculated for children and adults. Dietary intake via fish consumption was 3.20–25.13 ng/kg of body weight (b.w.) for children and 1.68–8.30 ng/kg b.w. for adults. Baltic fish caught along Polish coastal areas are a significant source of PFASs, especially for children.

**Supplementary Information:**

The online version contains supplementary material available at 10.1007/s11356-023-26626-w.

## Introduction

Persistent organic pollutants (POPs) are a group of compounds that are widely distributed, degradation resistant, bioaccumulative, able to be transported through the air, and toxic to humans and wildlife (Stockholm Convention 2001). Perfluorooctane sulfonic acid (PFOS) was listed at the Stockholm Convention in 2009 and perfluorooctanoic acid (PFOA) was in 2019. Perfluoroalkyl substances (PFASs) have been produced since 1950 and used in various applications such as surfactants, food contact materials, and firefighting foams (Buck et al. 2011; Fujii et al. 2013; Lindstrom et al. 2011; Prevedouros et al. 2006; Wang et al. 2014). In 2016 PFOA was classified by the International Agency for Research on Cancer (IARC) to group 2B as possibly carcinogenic to humans (IARC 2016). Pollutants in this group are responsible for the decreased response of the immune system to vaccinations (EFSA 2020). They are also related to immunotoxicity, developmental toxicity, and thyroid disorders (Bloom et al. 2010; Johnson et al. 2014; Mogensen et al. 2015).

As a result of the European Food Safety Authority (EFSA) risk assessment in 2020, a tolerable weekly intake (TWI) was established for the compounds that are responsible for approximately half of the exposure to PFASs (PFOA, PFOS, perfluorononanoic acid (PFNA), and perfluorohexane sulfonic acid (PFHxS)) of 4.4 ng/kg body weight (b.w.) (EFSA 2020). To protect human health and the environment, the European Union prohibited the use and manufacture of PFOS, PFOA, and their salts (EU 2019, 2020). Human exposure to PFASs occurs mainly through food and water, but inhalation and dust ingestion may also play a role (EFSA 2020; Enault et al. 2015; Ericson et al. 2008; Fraser et al. 2013). The source of PFASs in an aquatic environment (and food fish habitat) might be atmospheric deposition, effluents coming from municipal wastewater treatment plants or leaching of waste landfills (Ahrens and Bundschuh 2014). According to EFSA data, fish-origin PFASs make up one of the largest constituents of the overall PFAS intake in the European population (EFSA 2020). Besides various persistent organic pollutants like polychlorinated dibenzo-p-dioxins and dibenzofurans (PCDD/Fs), polychlorinated biphenyls (PCBs), polybrominated dibenzo-p-dioxins (PBDDs), polybrominated dibenzofurans (PBDFs), and polybrominated diphenyl ethers (PBDEs), PFASs were also found in fish species from the Baltic Sea (Fliedner et al. 2020; Kumar et al. 2022; Mikolajczyk et al. 2021; Zacs et al. 2013). Data from Finland and Sweden indicate that levels of PFASs found in Baltic fish can lead to exceedance of the TWI for fish consumers (Faxneld et al. 2016; Kumar et al. 2022).

The aim of our study was to investigate the PFAS contamination status of the Baltic fish species caught in the International Council for the Exploration of the Sea (ICES) zones located along the Polish coast. To assess the risk for fish consumers, the intake of PFASs which resulted from various fish consumption scenarios was calculated and then compared with the TWI.

## Materials and methods

### Sample collection

**Fig. 1:**
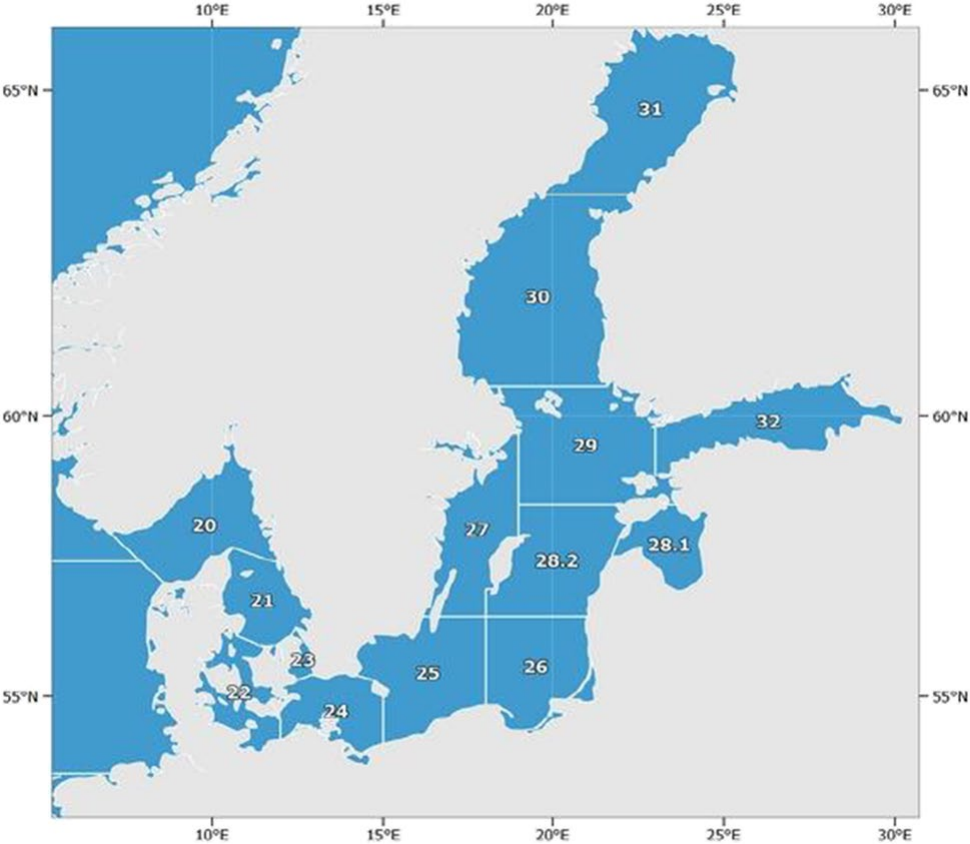
Map of the Baltic Sea showing the ICES areas (https://www.fao.org/fishery/en/area/27)

Altogether 63 fish samples were collected from three ICES Baltic fishing areas (24, 25, and 26) (Fig. 1) by the Veterinary Inspectorate (Table 1).

**Table 1:** Analyzed fish species details

Fish species	ICES areas	n	Weight g	Size cm
salmon (*Salmo salar*)	25, 26	10	1742 – 3500	53 – 70
trout (*Salmo trutta mtrutta*)	24, 26	10	1120 – 1932	38 – 57
sprat (*Spratus spratus balticus*)	24, 25, 26	20	4 – 26	4 – 20
herring (*Clupea harengus membras*)	24, 25, 26	20	9 – 229	10 – 30
cod (*Gadus morhua callarias*)	25, 26	3	539 – 1013	38 – 49

The Baltic Sea fish species that were chosen for investigation were salmon (*Salmo salar*), sea trout (*Salmo trutta* m. *trutta*), cod (*Gadus morhua callarias*), sprat (*Sprattus sprattus balticus*), and herring (*Clupea harengus membrus*). Bigger fish (salmon, trout, and cod) were tested individually and smaller fish were pooled. Salmon, trout, cod, and herring were tested through a slice of the fish muscles from backbone to belly in the middle part of the fish, while sprat were tested as whole fish. Sampling was performed in accordance with provisions of Regulation (EU) 2017/644.

### Analytes of interest, standards, and reference materials

The following compounds were investigated: PFOS, PFOA, PFNA, PFHxS, perfluorobutanesulfonic acid (PFBS), perfluoropentanesulfonic acid (PFPeS), perfluorohexanoic acid (PFHxA), perfluoroheptanoic acid (PFHpA), perfluorodecanoic acid (PFDA), perfluoroundecanoic acid (PFUnDA), perfluorododecanoic acid (PFDoA), perfluoroheptanesulfonic acid (PFHpS), perfluorotridecanoic acid (PFTrDA), and perfluorotetradecanoic acid (PFTeDA).

The following isotopically labeled analogs were used: sodium perfluoro-1-[1,2,3,4-^13^C_12_] octanesulfonate, perfluoro-n-[1,2,3,4-^13^C_12_] octanoic acid, perfluoro-n-[1,2,3,4,5-^13^C_12_] nonanoic acid, sodium perfluoro-1-hexane[^18^O_2_]sulfonate, perfluoro-n-[1,2,3,4,6-^13^C_12_] hexanoic acid, sodium perfluoro-1-[2,3,4-^13^C_12_] butanesulfonate, perfluoro-n-[1,2,3,4-^13^C_12_] heptanoic acid, perfluoro-n-[1,2-^13^C_12_] decanoic acid, perfluoro-n-[1,2,3,4,5,6,7-^13^C12] undecanoic acid, perfluoro-n-[1,2-^13^C_12_] dodecanoic acid, and perfluoro-n-[1,2-^13^C_12_] tetradecanoic acid. Sodium perfluoro-[1,2,3,4,5,6,7,8-^13^C_12_] octanesulfonate was used as an internal recovery standard. All standards were purchased from Wellington Laboratories Inc. (Ontario, Canada).

As reference material, certified IRMM-427 pike perch was purchased from the European Commission Joint Research Centre (JRC, Geel, Belgium).

### Sample preparation, extraction, purification, and detection

Fish muscles were homogenized and freeze-dried. One gram of the lyophilized sample was fortified with labeled standards at a concentration of 1 ng. Ten milliliters of 0.01 M methanol (LGC Standards, Wesel, Germany) and potassium hydroxide (POCH, Gliwice, Poland) were used for extraction. Then the sample was passed through an Oasis WAX solid phase extraction (SPE) cartridge (150 mg, 6 mL) (Waters Corp., Milford, MA, USA) and ENVI Carb Solid Phase cartridge (500 mg, 6 mL) (Supelco, Bellefonte, PA, USA). Compounds from the Oasis WAX cartridges were eluted using a mixture of methanol and ammonia (99.5/0.5; v/v). After passing the sample through the ENVI Carb cartridge, compounds were eluted by a methanol and acetic acid (80/1; v/v) solution. Recovery internal standard was added before liquid chromatography–tandem mass spectrometry (LC–MS/MS) analysis.

A Triple Quad 7500 mass spectrometry system (Sciex, Framingham, MA, USA) was used for quantification operated in the negative electrospray ionization (ESI −) mode. For chromatographic separation, a Gemini C18 column (3 μm, 50 × 2.0 mm) equipped with a guard column (Phenomenex, Torrance, CA, USA) was used. Ammonium acetate aqueous solution (20 mM) (A) and methanol (B) were the mobile phases. The gradient program of the mobile phase used was as follows: 25% B at 0 min, 30% B at 2 min, 98% B at 7–11 min, and 30% B until the end of the program at 20 min. The injection volume was 10 μL and the flow rate was 0.6 mL/min. Acquisition and processing were achieved using the Sciex OS software. All PFASs were confirmed by at least two MS/MS transitions in MRM mode. Linear L-PFOS and branched Br-PFOS were quantified using the linear standards. Limits of quantification (LOQ) and recovery ranges for individual PFASs are presented in Table S1 in the supplementary materials.

### Results presentation and statistical analysis

Concentrations of each compound are expressed in µg/kg of wet weight (w.w.). Sum concentrations are given as upper bound (UB) concentrations, whereby the concentration of each non-quantified congener (below the LOQ) was replaced with the LOQ, and as lower bound (LB) concentrations, whereby the concentration of each congener below the LOQ was replaced with the value 0.

To verify the normal distribution of the data, the Shapiro–Wilk test was used. Differences between experimental groups were verified using the Kruskal–Wallis test (at p ≤ 0.05). Correlations between the occurrences of individual PFASs were studied using Spearman’s correlation analysis. Statistical analyses were performed using Statistica software version 10, (StatSoft Inc., Tulsa, OK, USA) at the significance level of 0.05 (p < 0.05).

### Quality assurance and quality control

The solid-phase extraction cartridges and all solvents were verified to be PFAS free before use. For every batch consisting of ten samples, a procedural blank was included. A solvent blank was run before LC–MS/MS analysis.

Certified IRMM-427 pike perch reference material was used with each batch of 10 routinely analyzed samples. The results of the reference material analysis are presented in Table S2 in the supplementary materials. Trueness was in the range of − 19% to + 15%. Quality was controlled externally by successful participation in proficiency testing organized by the European Union Reference Laboratory for Halogenated Persistent Organic Pollutants in Feed and Food (EURL, Freiburg, Germany).

### Dietary intake

To characterize the potential health risk associated with ∑4 PFAS intake, doses ingested with fish were compared with the TWI of 4.4 ng/kg b.w. per week. The calculations were performed for an adult of 70 kg body weight and 3–10-year-old children of 23.1 kg (EFSA 2012) consuming each of three different portions of fish per week: consumption of the weekly average in Poland of 63 g, consumption of a 100 g portion of fish, and consumption of a 200 g portion per week (Statistics Poland 2021).

## Results and discussion

### Sum of 14 PFAS and 4 PFAS concentrations

**Fig. 2:**
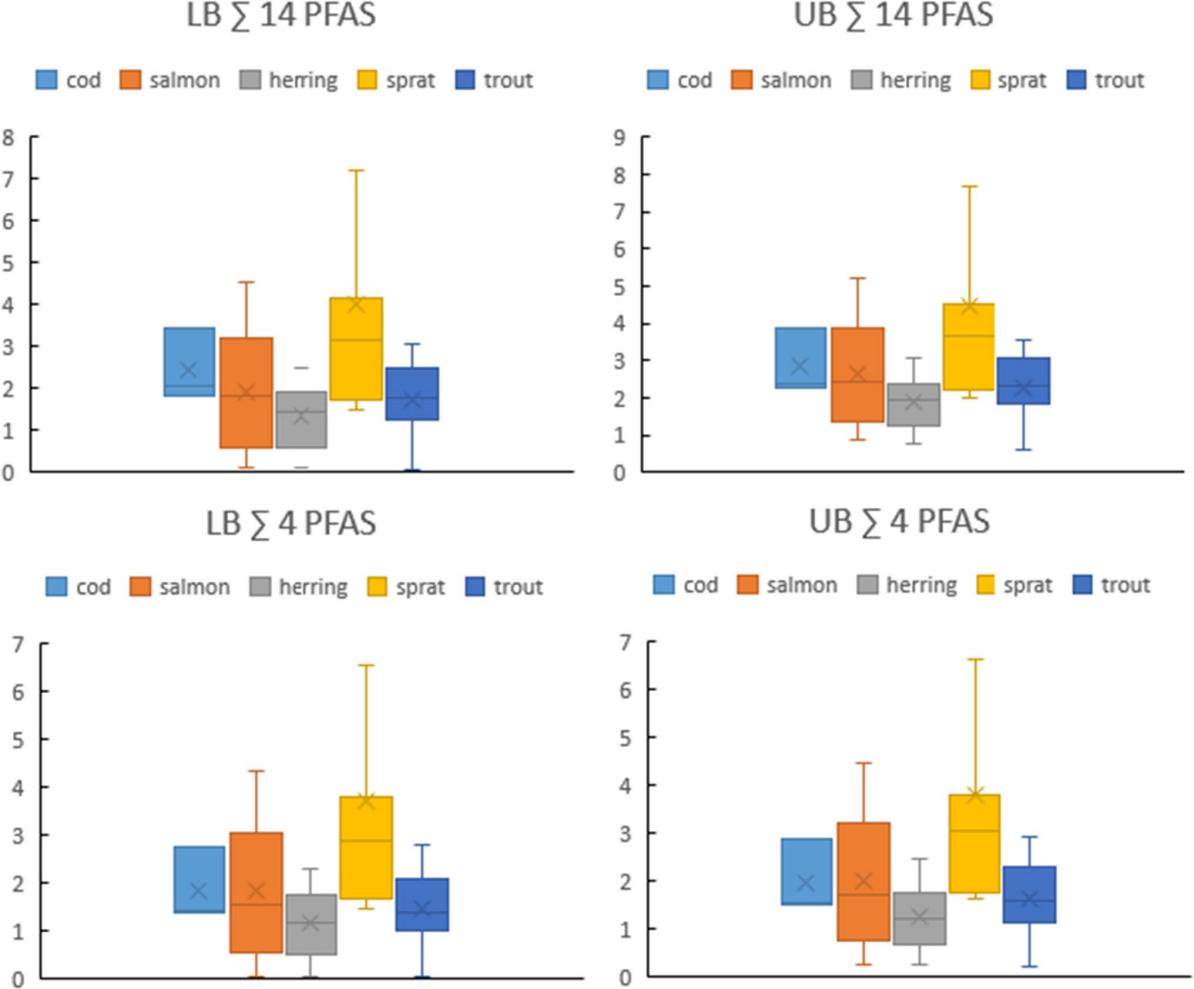
Box-plot of ∑14 PFASs and ∑4 PFASs concentrations (µg/kg w.w).

The median concentrations of ∑14 PFASs and ∑4 PFASs are presented in Fig. 2. The LB concentrations of ∑14 PFASs were as follows: 3.54 µg/kg w.w. in sprat, 2.15 µg/kg w.w. in cod, 2.10 µg/kg w.w. in salmon, 2.03 µg/kg w.w. in trout and 1.74 µg/kg w.w. in herring. The differences between species were not statistically significant (p > 0.05). Regarding the median LB of ∑4 PFASs, the difference was statistically significant only between sprat and herring (p < 0.05). The median LB and UB concentrations did not differ by more than 17% for ∑14 PFASs and 13% for ∑4 PFASs. The median LB concentration of ∑4 PFASs constituted 82% of that of ∑14 PFASs for sprat, 73% for salmon, and 66–69% for herring, trout, and cod. The highest median LB for ∑4 PFASs from all analyzed species was in sprat (12.76 µg/kg w.w.) and in this sample, PFOS constituted 93% of the sum.

### Individual PFASs concentrations

Concentrations of PFAS are presented in Tables S3 and S4 in the supplementary material. PFOS was most frequently detected in all analyzed samples regardless of the fish species, which was similar to the outcome of other research investigating PFAS fish contamination (Goodrow et al. 2020; Junttila et al. 2019; Kumar et al. 2022; Zafeiraki et al. 2019). The median concentrations of L-PFOS were as follows: 1.91 µg/kg w.w. in sprat, 1.30 µg/kg w.w. in salmon, 1.23 µg/kg w.w. in trout, 0.95 µg/kg w.w. in cod, and 0.72 µg/kg w.w. in herring (Table S3). In sprat samples, the median concentration of Br-PFOS was 0.55 µg/kg w.w., while the concentrations in the other species were in the range of 0.22–0.28 µg/kg w.w. (Table S3). Linear PFOS was mainly produced using telomerization processes, whereas a mixture of linear and branched acid was produced in the electrochemical fluorination (Buck et al. 2011). The average proportion of L-PFOS in the total PFOS (Br- and L-) was the highest in salmon (89%) and trout (87%), exceeding those in the rest of the species (75–80%). The investigation established that the highest level of L-PFOS was in a sprat sample (9.16 µg/kg w.w.) and this sample also had the highest levels of Br-PFOS (2.65 µg/kg w.w.) and other PFASs: PFHxS, PFNA, PFHpS, PFUnDA, and PFTrDA. No statistically significant differences in PFOS concentrations between species were revealed. The mean PFOS level in herring samples from this study of 0.81 µg/kg w.w. was higher than that in samples of this species caught in waters around the UK and European coasts of the North Atlantic, the mean of was 0.59 µg/kg w.w, while sprat from our study were less contaminated than those from the UK, having a mean 2.63 µg/kg w.w. concentration against the mean of 3.94 µg/kg w.w in the UK samples (Fernandes et al. 2018). The concentration of PFOS reported in herring from the northern part of the Baltic Sea along the Finnish coast (ICES zones 29, 30, and 32) was substantially higher at a median 1.90–3.40 µg/kg w.w. than our median concentration of 0.72 µg/kg w.w., but sprat from those same zones were similarly contaminated with 1.30–2.50 µg/kg w.w. PFOS concentrations to those in this study, with a median 1.91 µg/kg w.w. (Kumar et al. 2022). Sprat caught along the Swedish coast (ICES 31) contained PFOS at the level of 0.45–0.50 µg/kg w.w., and in ICES 25, their contamination was 0.64–0.67 µg/kg w.w, which was lower than our result of 0.81 µg/kg w.w. However, in ICES 27 the concentrations were significantly higher at 3.1–3.3 µg/kg w.w. (Faxneld et al. 2014). The median level of PFOS in salmon of 1.3 µg/kg w.w. was two- to three-fold lower than those in salmon caught along the Finnish coast (ICES 30–32) of 2.7–4.3 µg/kg w.w. (Kumar et al. 2022). Schultes et al. revealed a rising trend of PFOS in cod caught south-east of Gotland between 1981 and 2013. Higher increases were found for the branched (4.9% per year) than the linear isomer (3.3% per year) (Schultes et al. 2020).

Perfluorooctanoic acid was not observed in any samples of trout, salmon, or cod but was detected in 25% of samples of herring and 35% of samples of sprat. Concentrations of PFOA in herring along the Swedish coast, which fell in a < 0.02–0.15 µg/kg w.w. range (Faxneld et al. 2014), were similar to our results from ICES zones 24–26, while along the Finnish coast, in ICES 29 and ICES 31, the median levels of 0.41 and 0.76 respectively were considerably higher than our median of 0.11 µg/kg w.w. (Kumar et al. 2022). Also, a higher mean 0.34 µg/kg w.w PFOA level in herring caught in waters around the UK and the European coasts of the North Atlantic was noted (Fernandes et al. 2018). Over a tenfold higher concentration in UK-caught sprat, in which 1.48 µg/kg w.w was found (Fernandes et al. 2018), contrasted starkly with the 0.12 µg/kg w.w. detected in the present research.

Perfluorohexane sulfonic acid was not detected in cod or trout and appeared only in one sample of salmon, but was detected in 25% of herring and in 55% of sprat. The occurrence of PFHxS was correlated with L-PFOS (r = 0.77, p < 0.05) in sprat but not in the rest of the species. These two compounds were reported by others to co-occur as a result of emissions from fluoropolymer plants or use of firefighting foams (Bach et al. 2017; McGuire et al. 2014). It is hard to compare our PFHxS data with others’, because the concentrations determined by us were lower than the LOQ of the methods used by researchers investigating the northern parts of the Baltic Sea.

Perfluorononanoic acid was found in one sample of salmon, in all three samples of cod, in 30% of trout samples, in 60% of herring samples, and in 80% of sprat samples. Our herring data show similarity in contamination by PFNA to those for samples from the Swedish coast, where < 0.03–0.15 µg/kg w.w was detected (Faxneld et al. 2014), but lower contamination than Finnish data show from ICES zones 29–32 stating a 0.27–1.90 µg/kg w.w. concentration range (Kumar et al. 2022).

Perfluorohexanoic acid occurred in all three cod samples, in 60% of salmon samples, in 50% of trout samples, in 50% of herring samples, and in 80% of sprat samples. It was not present in contamination reported from the Finnish Baltic coast (Junttila et al. 2019; Kumar et al. 2022). It was also absent along the Swedish coast, but the method LOD of 0.53 µg/kg w.w. in the Swedish Baltic research was markedly higher than our LOQ (de Wit et al. 2020).Perfluorodecanoic acid was not detected in salmon or trout but was found in 10% of samples of herring, in 20% of samples of sprat, and in cod. In the northern part of the Baltic Sea along the Finnish coast, it was detected more frequently: in 61% of samples (Kumar et al. 2022). A higher concentration of PFDA in herring of 0.76 µg/kg w.w. was noted in the Bothnian Bay (Kumar et al. 2022).

Perfluoroundecanoic acid was present in all cod samples, in 60% of salmon samples, in 80% of trout samples, in 30% of herring samples and in 65% of sprat samples. Comparable PFuDA concentrations were found in herring catches all along the Swedish coast, where 0.03–0.13 µg/kg w.w. concentrations were yielded (Faxneld et al. 2014), and the Finnish coast, herring there being contaminated with a mean 0.11 µg/kg w.w. concentration (Junttila et al. 2019). A much higher level similar to that of PFDA was observed in herring from the Bothnian Bay, and it was 0.69 µg/kg w.w (Kumar et al. 2022).

An upward trend of PFHxS, PFNA, PFDA, PFUnDA, and PFDoA concentrations in cod muscles between 1981 and 2013 was found in the southeastern part of Gotland (Schultes et al. 2020). The highest increases were in PFDoA and averaged 7.3% per year, slightly lower rises occurred in PFUnDA of an average 7.2% per year, and other notably high increments emerged in PFDA, the concentration of this PFAS rising by 5.9% per year. In this study, in contrast, PFDoA was not detected in any samples regardless of the fish species (Table S4).

Perfluorotetradecanoic acid was contained by one cod sample, one salmon sample, 70% of herring samples, 55% of sprat samples, and 60% of trout samples. It was below the LOQ at 0.37 µg/kg w.w. in fish from ICES 29, 30, and 32 along the Finnish coast (Junttila et al. 2019; Kumar et al. 2022), and very low < 0.05–0.07 µg/kg w.w. concentrations of it were found in herring caught along the Swedish coast (Faxneld et al. 2014).

Perfluorotridecanoic acid contaminated two samples of cod, one sample of salmon, 15% of herring samples, 30% of sprat samples, and 40% of trout samples. The concentrations that we found were close to those obtained in Finnish research, in which all samples were below the LOQ at 0.36 µg/kg w.w (Kumar et al. 2022), and to those in Swedish data, where it was 0.03–0.20 µg/kg w.w (Faxneld et al. 2014).

Perfluoroheptanesulfonic acid was detected only in two samples of sprat. This compound was not detected in herring along the Finnish coast (Junttila et al. 2019; Kumar et al. 2022). No samples gave detection of PFBS, PFHpA, PFPeS, or PFDoA, regardless of fish species (Table S3). Similarly to our results, PFHpA was not found along the Finnish Baltic coast (Kumar et al. 2022).

Perfluoroalkyl substance bioaccumulation by fish is influenced by the substances’ presence in sediments and surface water as well as the length of the chain. According to Goodrow et al. long-chain PFASs predominate in fish and sediments whereas short-chain variants do in surface water (Goodrow et al. 2020). The profile of fish contamination (its fingerprints) might be used as the identification of the contamination source; the profile in the liver is more appropriate for this than the profile in muscles (Langberg et al. 2022). According to Norwegian researchers, when PFOS and other perfluoroalkanesulfonic acids dominate in the liver of fish, firefighting foams are considered the source. When the contamination fingerprints point most to long-chained perfluoroalkyl carboxylic acids, long-range atmospheric transport and production of paper products are the probable sources, the former when ∑ PFASs is low and the latter when the sum is high with a high PFOS percentage. Setting our data against that background, it is hard to conclude definitively what the source of the contamination of the fish analyzed by us from ICES zones 24–26 was, but the paper industry can be considered as one of them and other sources can be mooted to also be involved.

### Percentage share of compounds in ∑14 PFASs and ∑4 PFASs

**Fig. 3:**
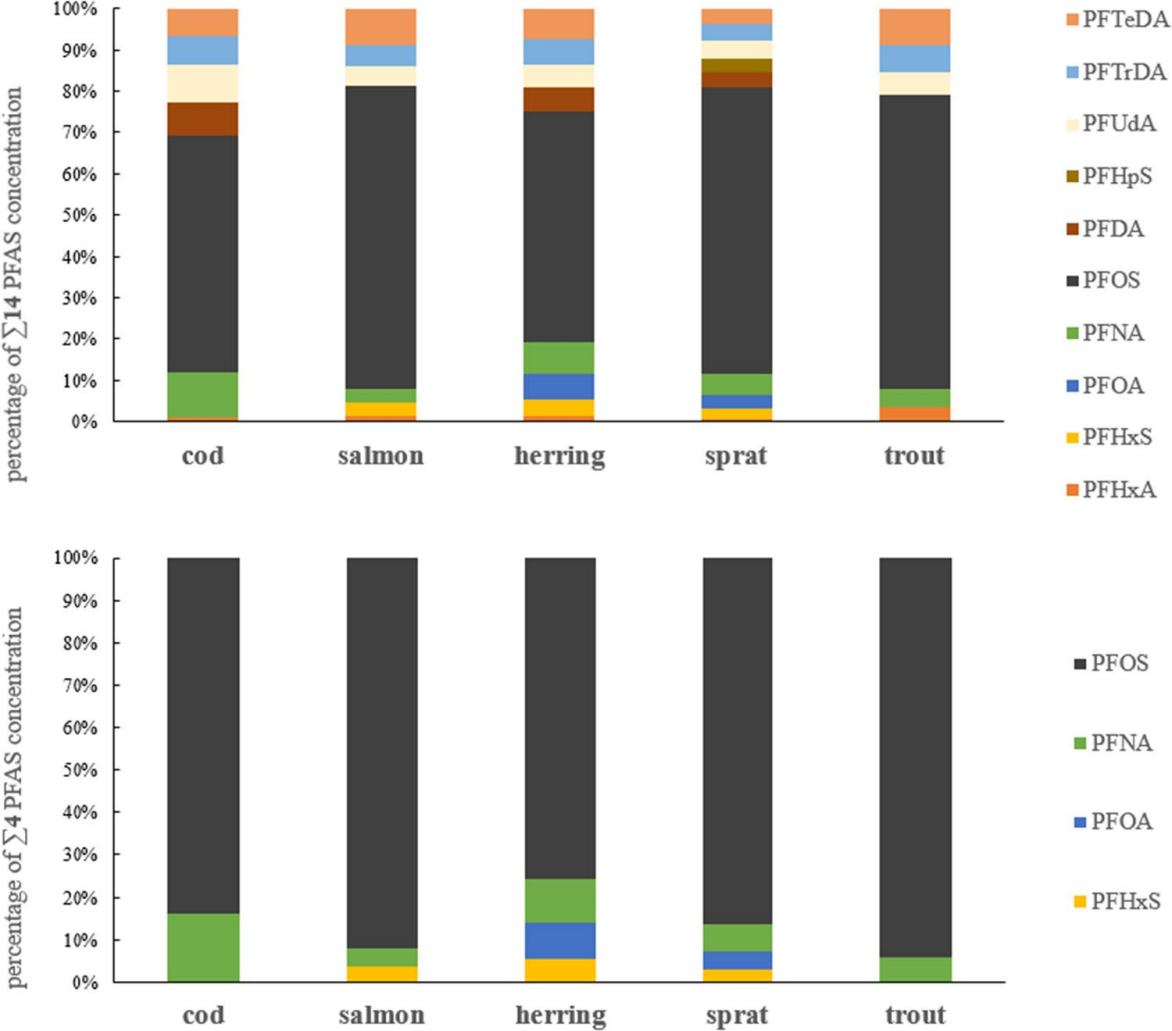
Percentage share of individual compounds in the LB concentrations of ∑14 PFASs and ∑4 PFASs

The sum of PFOSs made the highest contribution to the ∑14 PFASs in the following order: it was 73% in salmon, 71% in trout, 69% in sprat, 57% in cod, and 56% in herring (Fig. 3). A high contribution of PFOS was also found not only in other Baltic ICES zones (Faxneld et al. 2014; Koponen et al. 2015; Kumar et al. 2022) but also in fish from, Korean lakes (Hung et al. 2018) and the sea around the UK and the European coasts of the North Atlantic (Fernandes et al. 2018), and in fish from Greece (Costopoulou et al. 2022) and the Netherlands (Zafeiraki et al. 2019). The rest of the analyzed compounds did not contribute more than 11%. Regarding ∑4 PFASs, the ∑PFOS constituent was between 76% in herring and 94% in trout, and the remaining compounds did not constitute more than 16%.

### Dietary intake

**Fig. 4:**
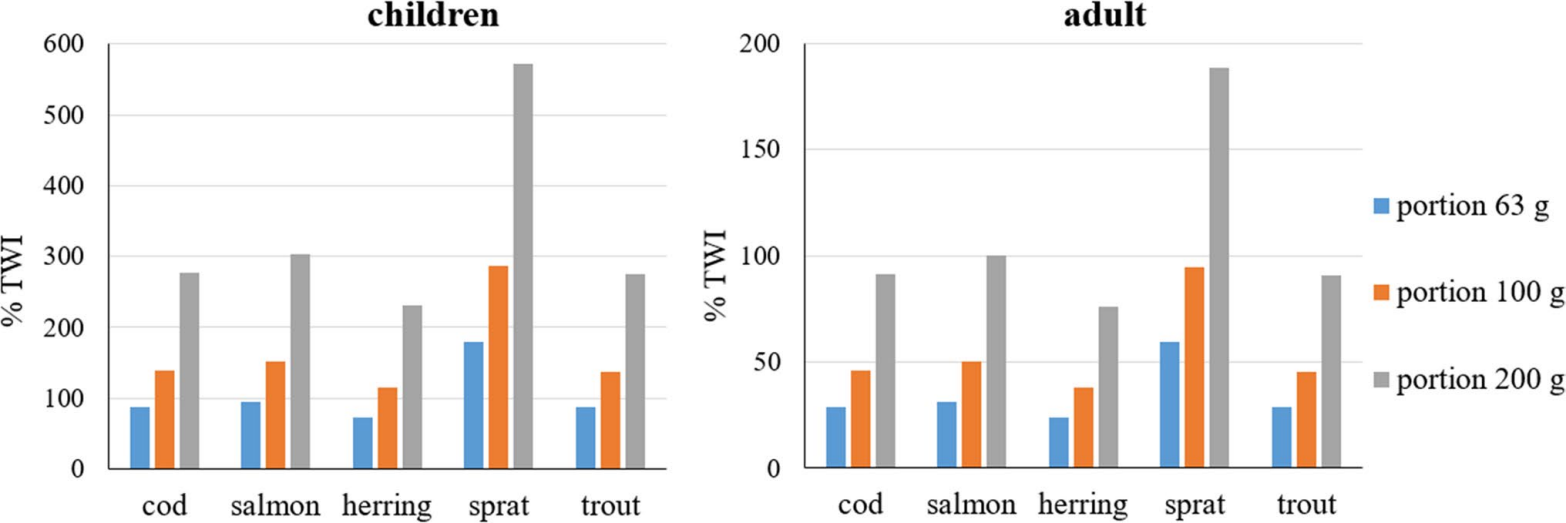
Estimated intake (ng/kg b.w.) of PFASs (PFOS, PFOA, PFNA, and PFHxS) with different portions of fish based on median lower bound concentration

The average consumption of fish in Poland published in the statistical yearbook includes consumption of all fish including freshwater fish and seafood, and therefore, calculations based on it produce overestimations. Such calculations are presented illustratively for the purposes of this work (Fig. 4) (Statistics Poland 2021). It is more appropriate to present intake via standardized 100 and 200 g portions of consumed fish (Fig. 4.). Calculations were made for median lower bound concentrations and assumed that all PFASs were absorbed after fish consumption. Dietary intake results are presented in Table 2. Taking into account the average consumption of 63 g, the estimated PFAS intake was 3.20–7.92 ng/kg b.w. for children and 1.06–2.61 ng/kg b.w. for adults (Table 2). Comparing these values with the TWI, consumption by adults of all Baltic fish did not lead to exceedance (Fig. 4), while for children consuming sprat, their intake would exceed the TWI by 80%. Consumption of other species contributed substantially to dietary intake, accumulating to 87–96% of the TWI. Consumption of 100 g of fish by adults exposed them to PFASs in an amount of 1.68–4.15 ng/kg b.w., which was still not an intolerable intake, but was close to being so in the case of sprat (94% of the TWI) (Fig. 4). In contrast and of concern was children’s exposure from consuming 100 g of fish to an amount of 5.08–12.57 ng/kg b.w., which was more than the TWI by margins in the range of 39%–186%.

**Table 2:** ∑4 PFAS intake

Fish species	Children			Adult		
	Fish portion					
	63 g	100 g	200 g	63 g	100 g	200 g
	∑4 PFAS intake ng/kg b.w					
Cod	3.85	6.11	12.22	1.27	2.02	4.03
Salmon	4.20	6.67	13.34	1.39	2.20	4.40
Herring	3.20	5.08	10.16	1.06	1.68	3.35
Sprat	7.92	12.57	25.13	2.61	4.15	8.30
Trout	3.83	6.07	12.15	1.26	2.00	4.00

The dietary intake via a 200 g fish portion for adults was estimated to be 3.35–8.29 ng/kg b.w. The tolerable weekly intake was exceeded only in the case of sprat, and eating this species exposed an adult to 189% thereof. However, consumption of other fish also burdened adults significantly, with 76–100% of the TWI. Consumption of a 200 g fish portion led to high exposure for children of 10.16–25.13 ng/kg b.w., which corresponded to a two- to six fold TWI exceedance. These data indicate that Baltic fish coming from ICES zones 24–26 are a significant source of PFASs for fish consumers, which is consistent with the indication from data from other Baltic regions (Falandysz et al. 2006; Kumar et al. 2022) and various parts of the world (Barbarossa et al. 2016; Costopoulou et al. 2022; EFSA 2020).

Cooking fish does not affect PFAS levels (Bhavsar et al. 2014; Taylor et al. 2019), while statistically significant increases in these levels were noted while frying or grilling it (Vassiliadou et al. 2015). For this reason, the cooking process has to be borne in mind while assessing risk to consumers.

Fish consumption was associated with high plasma PFAS levels in reproductive-aged women (Zhou et al. 2019). Trans placental transfer is the main source of PFAS exposure for a fetus (Apelberg et al. 2007), while breastfeeding is also a factor for newborns (Cariou et al. 2015). Since consumption of the recommended Baltic fish portion of 200 g per week can lead to TWI exceedance, and taking into account that other POPs are present in Baltic fish such as dioxins and PCBs (Mikolajczyk et al. 2021), appropriate advisories are needed for pregnant women, breastfeeding women, and children.

## Concluding remarks

The research conducted revealed high contamination of Baltic fish by PFASs. The differences in ∑14 PFAS concentrations between species were not statistically significant. Of all 14 analyzed PFASs, the isomer which was most responsible for contamination transpired to be PFOS. The high contamination of Baltic fish may lead to fish consumers, and particulary children, exceeding the TWI. These data indicate that frequent Baltic fish consumers are at an elevated health risk, and appropriate advisories are needed for pregnant women, breastfeeding women, and children.

## Supplementary Information

Below is the link to the electronic supplementary material.

Supplementary file1 (DOCX 23 KB)

## Data Availability

The data are available in supplementary.
