# Supplementary material for: Perfluoroalkyl substances in Baltic fish – the risk to consumers
Source: Environ Sci Pollut Res Int. 2023 Apr 3;30(21):59596–605. doi: 10.1007/s11356-023-26626-w (PMC10163105; doi:10.1007/s11356-023-26626-w)
Supplement: Supplementary file 1 — Supplementary file1 (DOCX 23 KB) [file 11356_2023_26626_MOESM1_ESM.docx]

**Perfluoroalkyl substances** **in Baltic fish- risk for consumers**

Table S1. Limits of quantifications (LOQ) and recoveries range.

| Compound | LOQ  (µg/kg of wet weight (w.w.)) | Recovery range of internal standard  (%) |
| --- | --- | --- |
| PFBS | 0.04 | 60 – 124 |
| PFHxA | 0.02 | 58 – 118 |
| PFHpA | 0.01 | 73 – 129 |
| PFHxS | 0.05 | 68 – 118 |
| PFOA | 0.07 | 60 – 126 |
| PFNA | 0.07 | 93 – 135 |
| PFOS | 0.01 | 87 – 116 |
| PFDA | 0.09 | 62 – 136 |
| PFPeS | 0.01 | - |
| PFHpS | 0.04 | - |
| PFUdA | 0.07 | 53 – 127 |
| PFDoA | 0.12 | 33 – 127 |
| PFTrDA | 0.10 | - |
| PFTeDA | 0.10 | 31 – 61 |

Table S2. Results of the certified reference material (IRMM-427 Fish tissue) analysis (µg/kg w.w.)

|  |  | **L-PFOS** | **Br-PFOS** | **PFNA** | **PFHxS** | **PFDA** | **PFuDA** | **PFDoA** | **PFTrDA** | **PFTeDA** |
| --- | --- | --- | --- | --- | --- | --- | --- | --- | --- | --- |
| 1 | | 16.6 | 0.96 | 0.09 | 0.10 | 1.36 | 0.85 | 1.00 | 0.60 | 0.49 |
| 2 | | 15.0 | 0.90 | 0.08 | 0.10 | 1.04 | 0.74 | 0.87 | 0.55 | 0.48 |
| 3 | | 14.1 | 1.01 | 0.09 | 0.10 | 1.37 | 0.85 | 0.94 | 0.70 | 0.51 |
| 4 | | 14.9 | 0.97 | 0.08 | 0.09 | 1.39 | 0.77 | 1.00 | 0.60 | 0.51 |
| 5 | | 13.8 | 1.06 | 0.08 | 0.09 | 1.21 | 0.79 | 0.93 | 0.58 | 0.49 |
| 6 | | 15.9 | 0.81 | 0.08 | 0.10 | 1.25 | 0.79 | 0.99 | 0.64 | 0.50 |
| **Certified value** | | **16.0** | **0.92** | **0.09** | **0.09** | **1.28** | **0.74** | **0.97** | **0.62** | **0.45** |

Table S3.Concentrations of PFOS, PFOA, PFNA and PFHxS expressed in µg/kg w.w..

| **Fish species** | | **n** | **L-PFOS** | **Br-PFOS** | **PFOA** | **PFNA** | **PFHxS** | **lower bound** |
| --- | --- | --- | --- | --- | --- | --- | --- | --- |
| sprat | median | 20 | 1.91 | 0.55 | 0.12 | 0.18 | 0.08 | 2.9 |
|  | range |  | 0.86 – 9.16 | 0.25 – 2.65 | <0.07 – 0.16 | <0.07 – 0.33 | <0.05-0.55 | 1.5 – 12.8 |
| herring | median | 20 | 0.72 | 0.25 | 0.11 | 0.13 | 0.07 | 1.17 |
|  | range |  | 0.06 – 2.04 | 0.00 – 0.39 | <0.07 – 0.17 | <0.07 – 0.30 | <0.05-0.09 | 0.06 – 2.30 |
| salmon | median | 10 | 1.30 | 0.26 | <0.07 | 0.07 | 0.06 | 1.54 |
|  | range |  | 0.05 – 3.76 | 0.03 – 0.54 | - | <0.07 – 0.07 | <0.05 – 0.06 | 0.05 – 4.36 |
| trout | median | 10 | 1.23 | 0.22 | <0.07 | 0.09 | <0.05 | 1.4 |
|  | range |  | 0.04 – 2.03 | 0.00 – 0.68 | - | <0.07 – 0.10 | - | 0.04 – 2.80 |
| cod | median | 3 | 0.95 | 0.28 | <0.07 | 0.24 | <0.05 | 1.41 |
|  | range |  | 0.91 – 1.84 | 0.23 – 0.60 | - | 0.20 – 0.32 | - | 1.4 – 2.8 |

Table S4.Concentrations of the rest PFAS expressed in µg/kg w.w..

| **Fish species** | | **n** | **PFBS** | **PFHxA** | **PFHpA** | **PFDA** | **PFPeS** | **PFHpS** | **PFuDA** | **PFDoA** | **PFTrDA** | **PFTeDA** |
| --- | --- | --- | --- | --- | --- | --- | --- | --- | --- | --- | --- | --- |
| sprat | median | 20 | <0.04 | 0.03 | <0.01 | 0.18 | <0.01 | 0.12 | 0.15 | <0.12 | 0.15 | 0.13 |
|  | range |  | - | <0.02 – 0.09 | - | <0.09 – 0.19 | - | <0.04-0.18 | <0.07-0.44 | - | <0.10-0.22 | <0.10-0.31 |
| herring | median | 20 | <0.04 | 0.03 | <0.01 | 0.10 | <0.01 | <0.04 | 0.10 | <0.12 | 0.11 | 0.13 |
|  | range |  | - | <0.02 – 0.04 | - | <0.09 – 0.11 | - | - | <0.07-0.17 | - | <0.10-0.13 | <0.10-0.23 |
| salmon | median | 10 | <0.04 | 0.03 | <0.01 | <0.09 | <0.01 | <0.04 | 0.10 | <0.12 | 0.11 | 0.19 |
|  | range |  | - | <0.02 – 0.05 | - | - | - | - | <0.07-0.17 | - | <0.10-0.11 | <0.10-0.19 |
| trout | median | 10 | <0.04 | 0.07 | <0.01 | <0.09 | <0.01 | <0.04 | 0.12 | <0.12 | 0.13 | 0.18 |
|  | range |  | - | <0.02 – 0.08 | - | - | - | - | <0.07-0.17 | - | <0.10-0.17 | <0.10-0.26 |
| cod | median | 3 | <0.04 | 0.02 | <0.01 | 0.17 | <0.01 | <0.04 | 0.20 | <0.12 | 0.15 | 0.14 |
|  | range |  | - | 0.02 – 0.03 | - | 0.14– 0.22 | - | - | 0.19-0.30 | - | <0.10-0.16 | <0.10-0.14 |
